# Worm in the Eye: A Case Report of Ocular Neurocysticercosis With Adherent Retinal Cyst

**DOI:** 10.7759/cureus.50194

**Published:** 2023-12-08

**Authors:** Kaanthi Rama, Vinay Jahagirdar, Akhileshwar Reddy R Ginnaram, Rahul Pottabathini, Vijaya Mandapalli

**Affiliations:** 1 Internal Medicine, Gandhi Medical College and Hospital, Hyderabad, IND; 2 Ophthalmology, Gandhi Medical College and Hospital, Hyderabad, IND; 3 Internal Medicine, University of Missouri Kansas City School of Medicine, Kansas City, USA; 4 Diagnostic Radiology, University of Arkansas for Medical Sciences, Little Rock, USA; 5 Ophthalmology, Guntur Medical College, Guntur, IND

**Keywords:** ophthalmoscopy, retinal cyst, helminth, ocular neurocysticercosis, tenia solium

## Abstract

Neurocysticercosis is caused by *cysticercus cellulosae*, the larval stage of *Taenia solium*, commonly referred to as the pork tapeworm. These larvae form cysts in several organs, including the brain, spinal cord, and eye. Neurocysticercosis is recognized by the World Health Organization as a public health issue and stands as the foremost preventable cause of epilepsy worldwide. Ocular neurocysticercosis refers to the concurrent involvement of the eyes and brain with cysticercus lesions. Neurological symptoms include focal deficits, intracranial hypertension, or cognitive decline. In the eye, the orbital type commonly presents with periocular swelling, ptosis, diplopia, restriction of ocular motility, or decreased vision. The ocular type shows signs of retinal detachment, a macular hole, and inflammation. A 45-year-old female presented with pain in his right eye with blurred vision for 15 days. On USG and MRI of the eye, a thin-walled lesion was noted. The brain showed a few calcified granulomas in the right parietal lobe on MRI. The left eye was normal. If left untreated, the cysts can lead to a severe inflammatory reaction in the eye, which may eventually lead to blindness. This blindness caused by cysticercus is preventable, and hence, early diagnosis and prompt medical or surgical treatment are essential.

## Introduction

Cysticercosis involving the central nervous system is referred to as neurocysticercosis. Designated as a neglected tropical disease by the World Health Organization, it is estimated that 50 million people around the globe have neurocysticercosis. It causes around 50,000 deaths annually and is the most frequent preventable cause of epilepsy worldwide [1]. Cysticercosis is a leading cause of preventable blindness in developing countries, where it is still endemic [2]. Eye involvement may be in the ocular or orbital regions, with extraocular muscular involvement being the most common type of presentation [3]. Ocular neurocysticercosis is a rare, uncommon presentation.

The diagnosis is confirmed by clinical, serological, and radiological investigations. Imaging includes high-resolution USG, CT, and MRI scans. Ocular bright scan (B-scan) reveals well-defined cysts with hyperechoic scolex [4]. We present a case of a 45-year-old female who presented with ocular neurocysticercosis with diminished vision for 15 days.

## Case presentation

A 45-year-old lady presented to urgent care with a painless diminution of vision in her right eye for 15 days. She had no significant past medical history and was not diabetic or hypertensive. There was no history of trauma to the eyes. She described the diminution of her vision as starting suddenly. She denied any photopsia, floaters, glare, itching, redness, photophobia, painful eye movements, doubling of vision, watering, discharge, or colored halos. She never had similar complaints in the past. A review of the systems was negative for headache, nausea, vomiting, weakness, and light-headedness. Vital signs were stable: she was afebrile, with a pulse of 70/min and a blood pressure of 120/80 mmHg.

On examination, her head posture was straight with no torticollis. Her face was symmetrical. Extraocular movements were not restricted. Both eyelids had normal positions, margins, lashes, and puncta with no restriction of movements. There was no redness or the presence of fleshy growths on the conjunctiva in both eyes. The cornea appeared normal. Bilateral irises were normal in color, pattern, and position. The pupil had a normal shape, size, and margin with intact pupillary reflexes. There were no focal deficits on the neurological examination.

The best-corrected visual acuity was 6/6 in the left eye and 6/24 in the right eye. On slit lamp examination, the anterior segment was unremarkable in both eyes: depth was normal, without any contents like hypopyon, hyphema, or aqueous flare. Applanation tonometry showed an intra-ocular pressure of 12 mmHg in the right eye and 13 mmHg in the left eye. All the above findings hinted at a pathology of the posterior segment of the eye.

**Figure 1:**
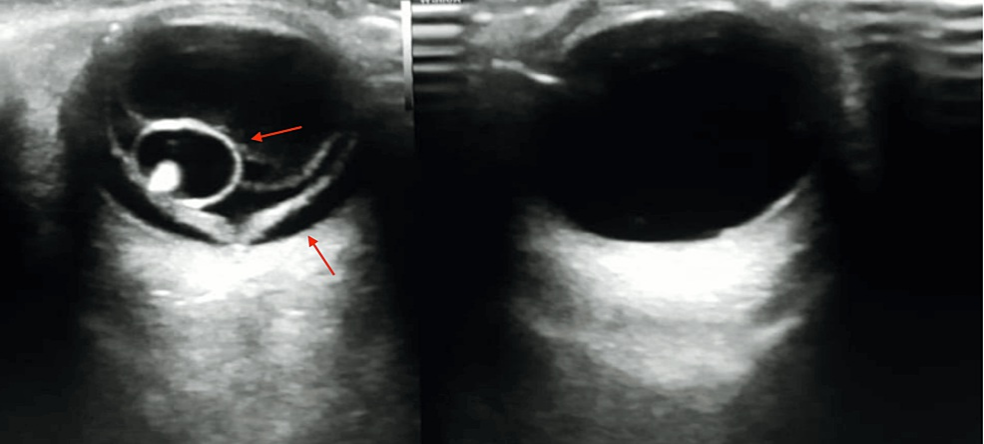
B-scan of the right eye showing cyst adherent to the retina

Indirect ophthalmoscopy on the right side disclosed an elevated, yellowish-white mass in the vitreous cavity. On the right eye ultrasound (Figure 1), an intravitreal cystic lesion with a hyperechoic focus adherent to the retina was identified, along with signs of exudative retinal detachment.

**Figure 2:**
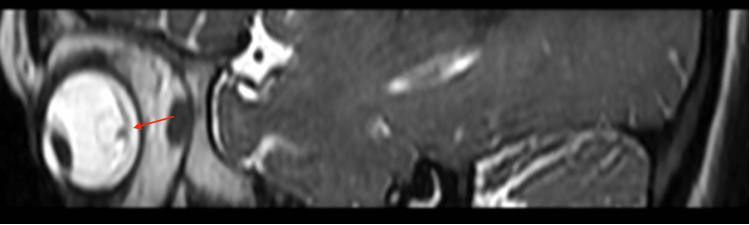
MRI of the lateral view of the eye showing cyst in the right eye

**Figure 3:**
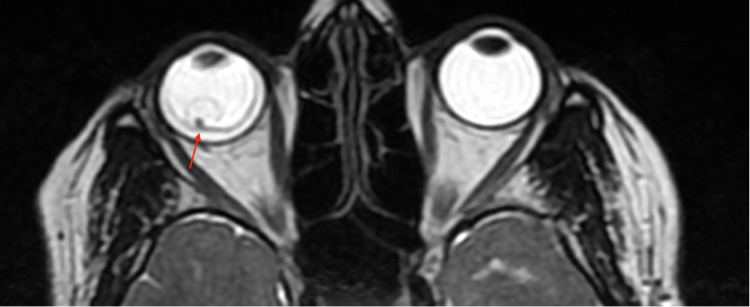
MRI of the T2W axial section showing cyst in the right eye

MRI of the right eye (Figure 2) and T2W axial section (Figure 3) showed a hypointense lesion enclosed by a thin wall. Intracranial calcified granulomas were noted in the right parietal lobe.

The patient’s history of sudden onset painless diminution of vision, examination findings of a normal anterior segment and mass in the vitreous cavity, combined with the imaging findings of a cystic lesion in the retina with calcified granulomas in the parietal lobe, all pointed toward neurocysticercosis with retinal detachment. The patient was eventually referred for surgical removal of the ocular cyst, where she received preoperative treatment with dexamethasone at a dosage of 5 mg/day for three days. Levetiracetam, at a dose of 1000 mg/day, was also administered for seizure prophylaxis. Upon examination six months post-surgical cyst removal, there was noted improvement in ambulatory vision with a best corrected visual acuity of 6/6 in the right eye, and the patient reported no new-onset seizures in the months following surgery.

The presence of a cystic lesion adherent to the retina with sub-retinal fluid and retinal detachment helps rule out other causes of sudden painless diminution of vision, such as central retinal artery occlusion, massive vitreous hemorrhage, ischemic and non-ischemic central retinal vein occlusion, and amblyopia. Differential diagnosis of posterior segment cysticercosis includes choroidal tumors and other parasitic infections like toxoplasmosis and rarely diffuse unilateral subacute neuro-retinitis.

## Discussion

Cysticercosis is caused by the larval form (metacestode) of the pork tapeworm, *Taenia solium*. Important risk factors associated with neurocysticercosis include poor sanitation, water contamination, and a lack of strict oversight over pig farms. Additionally, low health literacy and constraints on hospital resources delay timely diagnosis [5].

Clinical syndromes associated with this larval helminth include neurocysticercosis and extraneural cysticercosis. Neurocysticercosis is seen more commonly in the brain parenchymal and less commonly extraparenchymal, including intraventricular, subarachnoid, spinal, and, like our current point of discussion, ocular disease.

Life cycle

Larval stages of the *Taenia solium* cestode parasite may infect pigs and occasionally humans through the inadvertent consumption of their eggs present in the feces of human carriers. Pigs and humans become infected by ingesting eggs or gravid proglottids. Humans are infected by ingestion of eggs spread directly from another tapeworm carrier, from the environment, or by autoinfection.

In the latter case, humans infected with adult *Taenia solium* can ingest eggs produced by that tapeworm, most likely by the adherence of eggs to the hands and subsequent spread from hand to mouth. Once eggs are ingested, oncospheres hatch in the intestine, invade the intestinal wall, and migrate to striated muscles as well as the brain and other tissues, where they develop into cysticerci. In humans, cysts may cause serious sequelae if they localize in the brain/neuronal structures, resulting in neurocysticercosis [6-7]. Cysts located in the brain result in neurocysticercosis; humans with cysticercosis are incidental dead-end hosts. The median incubation period before the onset of symptoms is 3.5 years [8-9].

During the manifestation of the parasite disease as neurocysticercosis, we see it in a few different phases. A viable phase, which is usually asymptomatic, typically persists for many years without issue. Mechanisms for parasite evasion of the host immune response postulate that the parasite releases substances that may interfere with lymphocyte proliferation and macrophage function, thereby inhibiting normal cellular immune defenses [10]. In addition, humoral antibodies are not capable of killing the parasite.

The cysticerci eventually degenerate, likely because they lose their ability to evade the host immune response [11]. The appearance of contrast enhancement on X-ray and CT and/or edema implies the presence of a host immune response against the parasite. In the setting of enhancing intraparenchymal lesions, the inflammatory response is frequently associated with seizures [12]. Ultimately, the cysticerci resolve or become nonviable calcified granulomas, as seen in our case; the presence of intraparenchymal brain calcifications is also associated with seizures. Seizures are a very frequent manifestation in patients with degenerative cysts. The mechanisms by which epileptic seizures originate in neurocysticercosis are the subject of much contention and discourse. They are likely to involve the processes of local inflammatory changes and the subsequent generation of reactive gliotic scars in their pathogenesis [10].

Cysticerci may occur simultaneously at more than one anatomic site. In addition, cysticerci at different stages in their natural history may be present simultaneously. In our case here, the patient was suffering from ocular cysticercosis, which is a variety of extraparenchymal neurocysticercosis. Extraparenchymal neurocysticercosis has been found to occur more commonly in adults than in children [10-13].

Patients with ocular cysticercosis can involve portions of the subretinal space, vitreous humor, anterior chamber, conjunctiva, or extraocular muscles. The presence of subretinal cysticerci leans toward neurocysticercosis, whereas the presence of cysticerci in the anterior chamber of the eye implies extraneural cysticercosis (orbital) [14]. Neuro-oculo-cysticercosis can coexist in up to 24% of the cases [15].

Symptoms may include impaired vision, recurrent eye pain, and diplopia. Inflammation around degenerating cysticerci can threaten vision by causing chorioretinitis, retinal detachment, or vasculitis. Many patients may be asymptomatic [16]. Ocular cysticercosis should be excluded by an ophthalmologic examination in all patients with neurocysticercosis prior to initiating antiparasitic therapy. Direct visualization of the parasite by funduscopic examination is pathognomonic for the diagnosis of cysticercosis [16].

The main theory for the presence of the parasite in the posterior section of the eye is via the short ciliary vessels, particularly the arterial supply. The supply to the macula is the most vascularized, as well as being anatomically less thick, which tends to predispose it to larval lodging in these instances. The larvae, after being unable to travel further along, entrench themselves in the subretinal space. Here, it perforates into the vitreous cavity, leading to many of the symptoms described above. During the course of this process, the adverse effects that may be caused include retinal detachment, a macular hole, or the development of an inflammatory response to the parasite [17].

As the cyst develops, it causes atrophic changes in the overlying retinal pigment epithelium. It may also occasionally cause exudative retinal detachment and focal chorioretinitis. The central retinal artery is the most likely route for cysticercosis involving the optic nerve head [17]. The diagnosis of cysticercosis is forged based on clinical symptoms, neuroimaging findings, and epidemiologic exposure [16-19]. Neuroimaging studies should include both CT and MRI, when feasible [20].

Direct visualization of the parasite by fundoscopic examination is pathognomonic for the diagnosis of cysticercosis [18]. Although ocular cysticercosis is relatively uncommon and many patients are asymptomatic, inflammation around degenerating cysticerci can threaten vision, particularly in the setting of antiparasitic therapy. Hence, ocular cysticercosis should be excluded by an ophthalmologist examination in all patients with neurocysticercosis prior to initiating antiparasitic therapy.

Serologic testing is to be completed for confirmatory evaluation in patients with presumed cysticercosis [16]. These results are most useful for patients with neuroimaging findings that are assumed to resemble neurocysticercosis but are not confirmatory. Patients with diagnostic neuroimaging findings can be initiated on treatment before serologic results are available [16].

The serologic test of choice is an enzyme-linked immunoelectrotransfer blot (EITB) using parasite glycoproteins performed on serum [14]. The sensitivity of EITB varies with the form of neurocysticercosis and the specimen. Testing serum is generally more sensitive than testing cerebrospinal fluid [15]. In patients with multiple parenchymal lesions, ventricular lesions, or subarachnoid lesions, the sensitivity of serum EITB is nearly 100% [20]. This is of limited use in patients with a single parenchymal lesion or calcifications only, as the sensitivity is poor here [20].

Single-enhancing or cystic lesions can be treated with albendazole and steroids. Patients with more than two cystic lesions should be treated with combination therapy with albendazole and praziquantel, although some physicians recommend surgical excision of the cysts as the treatment of choice to avoid inflammatory reactions due to toxins released by dying larvae [15].

## Conclusions

Cysticercosis is a complex parasitic infection with diverse clinical presentations, and ocular cysticercosis warrants careful evaluation to ensure timely diagnosis and management. Comprehensive assessment, including funduscopic examination and serologic testing, is crucial in providing the best care for affected individuals.
